# Monitoring the West Nile virus outbreaks in Italy using open access data

**DOI:** 10.1038/s41597-023-02676-0

**Published:** 2023-11-07

**Authors:** Marco Mingione, Francesco Branda, Antonello Maruotti, Massimo Ciccozzi, Sandra Mazzoli

**Affiliations:** 1 Dept. of Political Sciences, Roma Tre University, Rome, Italy; 2 Institute of Applied Computing “M. Picone” (IAC-CNR), Rome, Italy; 3 Unit of Medical Statistics and Molecular Epidemiology, University Campus Bio-Medico of Rome, Rome, Italy; 4 Dept. of Law, Economics, Politics, and Modern Languages, LUMSA University, Rome, Italy; 5 STDs Centre, Santa Maria Annunziata Hospital, Florence, Italy

**Keywords:** Databases, Infectious diseases

## Abstract

This paper introduces a comprehensive dataset on West Nile virus outbreaks that have occurred in Italy from September 2012 to November 2022. We have digitized bulletins published by the Italian National Institute of Health to demonstrate the potential utilization of this data for the research community. Our aim is to establish a centralized open access repository that facilitates analysis and monitoring of the disease. We have collected and curated data on the type of infected host, along with additional information whenever available, including the type of infection, age, and geographic details at different levels of spatial aggregation. By combining our data with other sources of information such as weather data, it becomes possible to assess potential relationships between West Nile virus outbreaks and environmental factors. We strongly believe in supporting public oversight of government epidemic management, and we emphasize that open data play a crucial role in generating reliable results by enabling greater transparency.

## Background & Summary

West Nile Virus (WNV) belongs to the *Flaviviridae* family, genus Flavivirus which is a single-stranded, positive sense RNA virus^1^, and was first discovered in a Ugandan woman in 1937^2^. This virus is one of the many Arboviruses (Arthropod-born viruses) that are maintained in nature principally through biological transmission between susceptible vertebrate hosts by haematophagous arthropods. Ticks and mosquitoes^3^ are the principal vectors but also other blood-sucking arthropod vectors can be involved in Arbovirosis, as for Toscana virus meningitis in Italy^4^. At present, there exist more than five hundred viruses classified as Arbovirus, which can cause illnesses in animals^3^ and about a hundred in humans, the majority belonging to the *Togaviridae*, *Flaviviridae* and *Bunyaviridae* viral families. Before 1990, sporadic cases and mild outbreaks of WNV occurred, except in Israel and France. After 1990 several outbreaks have been observed in Algeria, Morocco, Tunisie, Italy, France, Romania, Israel and Russia, with neurological complications and deaths. In the summer of 1999, a New York cluster with its genomic sequence demonstrated the Israeli origin of the strain^1^. It is unknown how the virus crossed the Atlantic Ocean with subsequent additional spreading in Canada, the USA, Mexico, the South American Caribbean Area, Venezuela, Chile, and Argentina. South Africa and the Western Hemisphere are other infection cluster zones. In Italy, WNV was first detected in Toscana back in 1998^5^. The regions of Emilia-Romagna and Veneto, which surround the Po River delta, were particularly affected. Since then, WNV has been detected every year in the country. To address this ongoing concern, an integrated surveillance plan for Arboviruses was initiated in the Northern Italy regions in 2008 and subsequently extended to cover the entire country^6,7^.

Various external factors may contribute to the spread of the virus, including climate change^8–10^, urbanization, ease of travel, and globalization^11^. Temperature anomalies, in particular, have been found to influence WNV transmission in Europe. They can alter the geographic range of vectors, the aerial migration routes of avian WNV hosts, and the pathogen life cycle^8^. Mosquitoes of the *Culex pipiens* are recognized as the main vector of WNV in Italy^12^. The mosquito’s life cycle lasts from one to four weeks and is highly sensitive to weather conditions, temperature, and fluctuations in rainfall^13^.

Infections in animals, especially birds and equids, have been widely reported in the literature. Among birds, crows and other wild birds are the most commonly infected species. WNV has also been detected in rescue centres during the cold season^14^. A mosquito can acquire the virus from an infected bird and subsequently transmit it to a susceptible horse several days later. Horses typically become ill three to fourteen days after exposure to an infected mosquito (incubation period), but they cannot infect humans. Common early signs of infection in horses include twitching of the muzzle and ears, frequent chewing, aggression, and fine muscle twitching, followed by progressive lack of coordination, weakness, and listlessness. Paralysis of the limbs, seizures, disorientation, coma, or death may occur. Differential diagnosis involves considering rabies, eastern/western encephalitis, and equine protozoal myeloencephalitis (see https://www.ksvhc.org/services/equine/internal-medicine/west-nile.html).

Early phylogenetic studies detected seven different WNV lineages^1^. This former genotyping has been lately updated thanks to the introduction of more advanced genetic methods. Based on biology, evolution, pathogenicity, and geographic distribution, WNV has been grouped into nine lineages^15^, lineage 1 (WNV-1) and 2 (WNV-2) remaining the most pathogenic: lineage 1 is subdivided into 3 sub-lineages, including sub-lineage 1a with African, European, and Middle Eastern isolates. Sub-lineage 1b includes WNV_*KUN*_ strains from Australasia and sub-lineage 1c, also known as lineage 5, isolated from India^15^. WNV lineage 1 and 2 are endemic in Italy and co-circulate, and WNV-1 has been associated with an increased risk of neuroinvasive disease, especially in 2022^16^. Lineage 7, instead, first isolated in 1968 in Koutango (Senegal) and later in Somalia, was demonstrated to be more pathogenic than other virulent strains. In humans, the 80% of reported infections are asymptomatic or subclinical, while the remaining 20% cause mild to severe symptoms. Specifically, three main pathological forms have been observed in various host species: neuroinvasive, cutaneous and gastrointestinal. The neuroinvasive form of WNV infection is the most severe form of the disease and it is reported in the 1% of humans and equids infections. The main symptoms are meningitis, encephalitis, flaccid paralysis^1,6^, with lesions including granulocytic meningitis, lymphoplasmacytic-histiocytic perivascular cuffing, and lymphoplasmacytic meningo-encephalomyelitis. Cutaneous manifestations of WNV infections have also been extensively documented in humans. They show up as an erythematous, maculopapular rash, punctate exanthem affecting the extremities and the trunk. On the other hand, gastrointestinal syndrome in humans due to WNV is characterised by diarrhoea, enteritis, gastritis, pancreatitis, and hepatitis; this WNV gastrointestinal form is reported in up to 30% of human cases^15^.

All these aspects, jointly with the risk associated with contracting the virus for both humans and animals, highlight the importance of monitoring and analyzing WNV^17–20^, as well as other diseases^21–23^.

This paper presents an open access spatio-temporal dataset, named WNVDB, which includes data on West Nile virus outbreaks that occurred in the Italian territory from September 2012 up to November 2022. WNVDB is not novel in the sense that smaller subsets of the data it includes have already been comprehensively analyzed in dedicated studies^24–27^, but focused on shorter time-windows.

Nonetheless, here we highlight that the release of this dataset yields three key advantages. First, a centralized and always up-to-date repository containing all data about WNV outbreaks on the Italian territory that can be freely and easily consulted, favouring research analyses. Second, the utilization of standardized definitions and protocols (e.g., Italian administrative divisions are identified by the same codes used by the Italian National Statistical Institute (ISTAT)) allows the users to join WNV data to other official sources of information flexibly. Third, the adherence to the FAIR (Findability, Accessibility, Interoperability, and Reusability) principles for data management and stewardship^28^. Its scope is however wider. Having a completely open and readily available dataset about WNV in Italy can be highly useful for several reasons:

**Research and analysis:** the dataset can serve as a valuable resource for researchers and scientists studying WNV and its impact on public health. They can use the data to analyze the patterns of WNV outbreaks while developing effective prevention and control strategies. The dataset can contribute to advancing knowledge in the field and improving our understanding of the virus.

**Monitoring and surveillance:** an open dataset allows for continuous monitoring and surveillance of WNV outbreaks. Public health authorities, researchers, and policymakers can regularly access the data to track the spread of the virus, identify areas of high risk, and implement timely interventions. This information can help in rapid response and proactive measures to prevent further transmission.

**Collaborative efforts:** open data fosters collaboration among researchers, institutions, and organizations working on WNV. By sharing a comprehensive dataset, different stakeholders can collaborate, share insights, validate findings, and collectively contribute to a better understanding of the virus. This collaboration can lead to more effective strategies for disease prevention and control.

**Public oversight:** making the dataset openly available supports public oversight of government epidemic management. It enables transparency and allows citizens, journalists, and advocacy groups to examine the data, evaluate the government’s response to WNV outbreaks, and hold authorities accountable. Open data promotes trust and public engagement in the decision-making processes related to public health.

**Improved modelling and predictive capabilities:** access to a complete and open dataset enhances the accuracy and reliability of disease modelling and predictive capabilities. Researchers can use the data to develop sophisticated models that can forecast WNV outbreaks, assess the impact of environmental factors, and guide resource allocation and preparedness efforts. Accurate models can help in early warning systems, resource planning, and targeted interventions.

In summary, having a complete open and readily available dataset on WNV in Italy promotes research, monitoring, collaboration, public oversight, and the development of effective strategies to combat the virus. It empowers stakeholders with valuable information to address the challenges posed by WNV and protect public health.

The remainder of the paper is organised as follows: the Methods section describes the data sources and the protocol implemented for the construction of the dataset. The Data Records section introduces our dataset and the various associated metadata files. Then, the Technical Validation section follows to verify the statistical consistency of the data (e.g., intra- and extra-record coherence). Finally, some discussion is provided to show the potential of the dataset for research purposes in the Usage Notes section.

## Methods

The main data sources for this study are the bulletins published in PDF format by the Italian National Institute of Health (ISS), in collaboration with Office V of the Ministry of Health’s General Directorate for Preventive Healthcare and the Research Centre for Exotic Diseases (Centro studi malattie esotiche - CESME) of the Istituto Zooprofilattico Sperimentale dell’Abruzzo e del Molise “Giuseppe Caporale” (IZS-AM). More precisely, the bulletins are the product of an integration of human and veterinary surveillance systems that are run across Italy at the national level, under the mandate of the Italian Ministry of Health, by two Institutions: the ISS, who takes care of the human epidemiology and hosts the national reference laboratory as well as the National Focal Point for Emerging and Vector Borne diseases for the European Centre for Disease Prevention and Control (ECDC); the IZS-AM, who coordinates surveillance in horses, birds and mosquitoes at the national level. A comprehensive description of the “National prevention, surveillance and response plan for arboviral diseases (PNA) 2020–2025” is available at https://www.statoregioni.it/media/2371/p-1-csr-rep-n-1-15gen2020.pdf.

The surveillance initiative started in 2012 following the enactment of *DGPRE 0012922-P-12/06/2012*, which initially focused on monitoring neuroinvasive infections in humans. However, it was subsequently expanded in 2017 to include animals, specifically mosquitoes, birds, and equids. Therefore, the available data includes information on WNV infections in humans from June 2012 and in animals from August 2017.

**Fig. 1:**
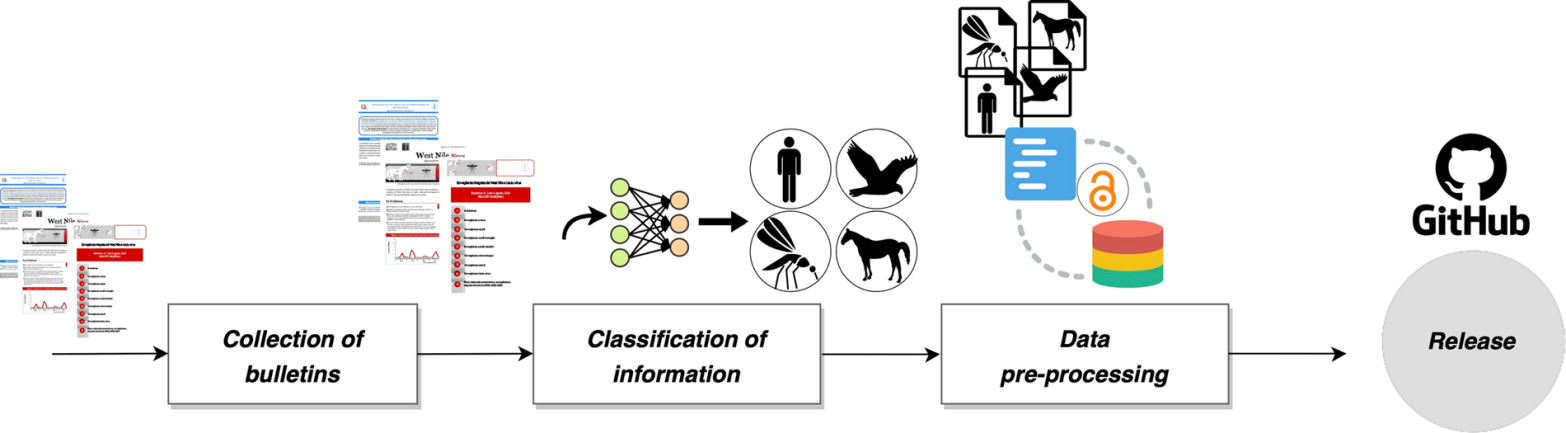
Schematic overview of the key steps to build the WNVDB open access dataset.

The data production process, covering from the digitalization to the release of WNVDB, is composed of four main steps that are summarized in Fig. 1. In the “collection of bulletins” step, we downloaded a total of 163 bulletins (up to November 2022) from the EpiCentro website (https://www.epicentro.iss.it/westnile/bollettino). After the download, in the “classification of information” step, we organized these bulletins according to the corresponding surveillance category. A standard bulletin typically consists of the following sections: (i) a textual description of human cases categorized by region and infection type; (ii) a section reporting human cases by province and infection type, including a table presenting neuroinvasive cases at the provincial level categorized by age group; (iii) a section providing information on verified outbreaks in equids at the regional and provincial levels in tabular form; (iv) separate sections describing cases in target species and wild birds; and (v) a final section reporting the number of mosquitoes caught and tested positive for the virus at the regional and provincial levels. The most challenging steps were (i) and (ii), which involved extracting information from unstructured textual data. The remaining steps were also not trivial but were facilitated by the use of an automatic tool called “Tabula” (https://tabula.technology/). This tool enabled the extraction and conversion of tables from the PDF files directly into data frames. Afterwards, a “data pre-processing” step was required to ensure the coherence and consistency of our dataset. Specifically, (i) a standardized procedure was adopted to encode geographic information following the ISTAT nomenclature, also including longitude and latitude, and (ii) weekly cases have been derived by calculating the first differences of the officially reported cumulative cases (“total_cases”) in the bulletins. Finally, the resulting dataset was released on a GitHub repository. The whole protocol was repeated for all bulletins, separately for each year, with a computational runtime varying from 30 minutes–for early bulletins with limited data–to several hours for more recent bulletins containing additional details such as infection type and/or host information. The code used to digitize the PDF bulletins is available (https://github.com/fbranda/west-nile/blob/main/extract_data.py), and we hope it could be useful as a general tool to extract structured information from unstructured documents.

## Data Records

**Fig. 2:**
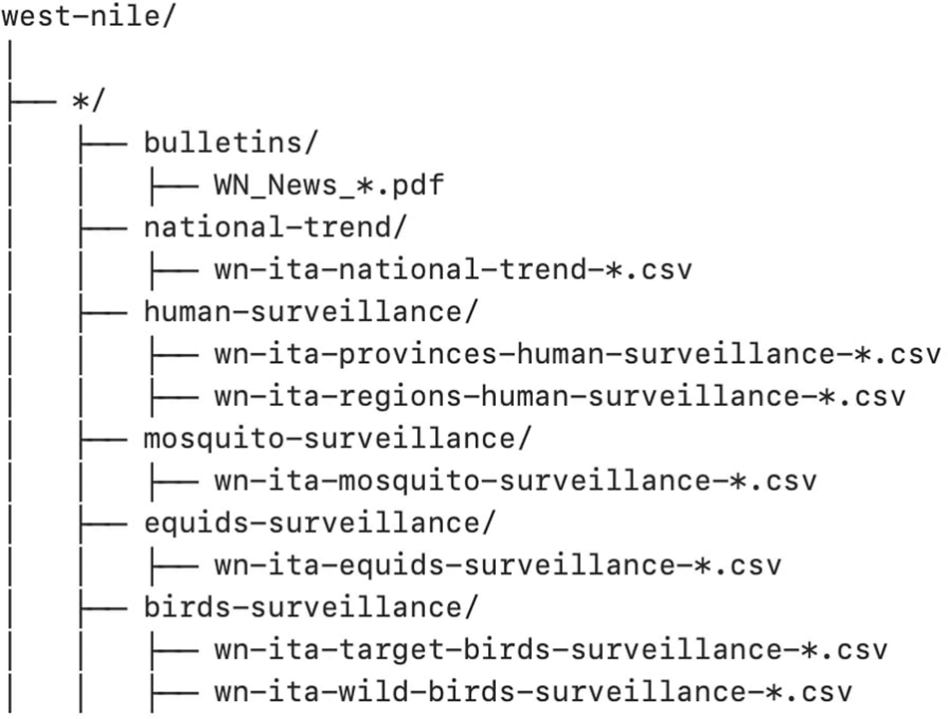
Schematic structure of WNVDB.

The static version of WNVDB that is described therein is hosted on Zenodo^29^. This dataset consists of one folder for each year, named from the first to the last available surveillance year. Each folder contains: (i) a subfolder called “bulletins” that includes all the PDF bulletins published in that year; (ii) a subfolder called “national-trend” that describes the national trend of WNV cases. In addition, depending on the amount of available information, the yearly folders may contain up to 4 subfolders called “*-surveillance”, where “*“ stands for humans (available for all years), equids, birds, and mosquitoes (available starting from 2017), each of which describes WNV cases per host at the regional and provincial levels. Figure 2 shows a schematic overview of the dataset structure, while a more detailed description of the folders is reported below.

### Bulletins

This folder includes the original PDF files with the data about veterinary and epidemiological surveillance of WNV as they are published by official sources. Each bulletin has been named as “WNV_News_yyyy_#.pdf”, where “yyyy” is the year when the bulletin has been published and “#“ identifies a sequential identification number.

### Trend at the national level

This folder contains data aggregated at the national level for WNV weekly and cumulative cases. Such data are organized into .csv files, called “wn-ita-national-trend-yyyy.csv” (where “yyyy” is the year of monitoring), whose structure is reported in Table 1. In particular, each file has 5 columns and a number of rows equal to *T* × *n*_host_, where *T* is the number of monitoring weeks and *n*_host_ is the distinct number of host (i.e., 0 = humans, 1 = equids, 2 = target birds, 3 = wild birds, 4 = mosquitoes) for which data are available.

**Table 1:** Structure of the file “wn-ita-national-trend-yyyy.csv” within the folder “national-trend”.

Variable	Description	Format
url_bulletins	Link to the bulletin in PDF format	String
data	Week reference date	yyyy-mm-dd
host	Name of host organism	String
new_cases	New cases (“total_cases” current date - “total_cases” previous date)	Numeric
total_cases	Cumulative number of cases	Numeric

### Human surveillance data

This folder contains data about WNV infections in humans, at both the regional and the provincial level, organized into two distinct .csv files, namely “wn-ita-regions-human-surveillance-yyyy.csv” and “wn-ita-provinces-human-surveillance-yyyy.csv” (where “yyyy” represents the year of monitoring). The former has a total of 9 columns and each row identifies the weekly number of cases by region and type of infection (i.e., neuroinvasive, fever, blood donor); the latter has instead a total of 13 columns and each row identifies the weekly number of WNV infections by province, age-class (i.e., ≤14, 15–44, 45–64, 65-74, ≥75) and type of infection. The structure of both csv files is reported in Tables 2, 3, respectively. Note that, from 2013 to 2017 only neuroinvasive cases were reported, while two more types of infection were added in the 2022 surveillance, namely symptomatic and asymptomatic. Also in 2022, cases imported from abroad were specifically recorded. We decided to include this information in the column “name_region” by adding one category for each country from which cases were imported, coding it as “imported from” +“country”. At present, only imported cases from Spain and Morocco were recorded.

**Table 2:** Structure of the file “wn-ita-regions-human-surveillance-yyyy.csv” within the folder “human-surveillance”.

Variable	Description	Format
url_bulletins	Link to the bulletin in PDF format	String
data	Week reference date	yyyy-mm-dd
code_region	Region 2-digit code	Numeric
name_region	Region name	String
lat	Latitude of the province	Numeric
long	Longitude of the province	Numeric
new_cases	New cases (“total_cases” current date - “total_cases” previous date)	Numeric
total_cases	Cumulative number of cases	Numeric
type_infection	Type of infection of reported cases	String

**Table 3:** Structure of the file “wn-ita-provinces-human-surveillance-yyyy.csv” within the folder “human-surveillance”.

Variable	Description	Format
url_bulletins	Link to the bulletin in PDF format	String
data	Week reference date	yyyy-mm-dd
code_region	Region 2-digit code	Numeric
name_region	Region name	String
code_province	Province 3-digit code	Numeric
name_province	Province name	String
abbreviation_province	Province 2-letter code	String
lat	Latitude of the province	Numeric
long	Longitude of the province	Numeric
age	Age of reported cases	String
new_cases	New cases (“total_cases” current date - “total_cases” previous date)	Numeric
total_cases	Cumulative number of cases	Numeric
type_infection	Type of infection of reported cases	String

Although in principle it is possible to aggregate data to the regional level starting from the data at province level, the fact that the surveillance plan slightly changed over time may cause some mismatch between the two files. This is unfortunately unavoidable in such a complex framework and the eventual correction of misaligned records would be the subject of future work. Here, we decided to keep both datasets separated, in compliance with the bulletins’ structure published by ISS, because the main goal was to faithfully report what is disseminated by official sources.

Overall, Italy counted a total of 1576 WNV infections, with a yearly average of 143 cases. However, all outbreaks were mild except the ones of 2018 and 2022, for which a total of 581 and 599 have been recorded, respectively. Excluding these two years from the computation yields a yearly average of only 44 cases, corresponding to < 1 case for each million residents. The yearly distribution by age does not substantially change over time. Infections in people aged ≤45 years are rarely recorded, while ≈22%, ≈27% and ≈46% of cases are recorded on average in 45–64, 65–74, ≥75 age classes each year. People over 75 are the ones mostly affected by symptomatic infections, as they are likely to have other age-related comorbidities.

### Mosquito surveillance data

This folder contains the weekly cases of WNV infections in mosquitoes at the province level. It includes the file “wn-ita-mosquito-surveillance-yyyy.csv” (where “yyyy” is the year of monitoring), the description of which is given in Table 4. Mosquitoes infections mostly hit the three regions of Northern Italy (Emilia-Romagna, Veneto and Lombardia) with some exceptions for Friuli-Venezia Giulia in 2020 and Piemonte in 2022. Indeed, ≈50% of West Nile mosquito infections are observed in Emilia-Romagna each year, ≈25% in Veneto, ≈8% in Lombardia, while the remaining ≈17% is spread out among Friuli-Venezia Giulia, Piemonte and Sardegna (only 4 cases in 2018 and 1 case in 2022).

**Table 4:** Structure of the file “wn-ita-mosquito-surveillance-yyyy.csv” within the folder “mosquito-surveillance”.

Variable	Description	Format
url_bulletins	Link to the bulletin in PDF format	String
data	Week reference date	yyyy-mm-dd
code_region	Region 2-digit code	Numeric
name_region	Region name	String
code_province	Province 3-digit code	Numeric
name_province	Province name	String
abbreviation_province	Province 2-letter code	Numeric
lat	Latitude of the province	Numeric
long	Longitude of the province	Numeric
new_cases	Total amount of new cases (“total_cases” current date - “total_cases” previous date)	Numeric
total_cases	Total number of cases	Numeric

### Equids surveillance data

This folder contains weekly cases of WNV infections in equids at the province level. Differently from the surveillance of other hosts, here it is also reported the number of animals in the outbreak and the number who died. The structure of the csv file included in this folder, namely “wn-ita-equids-surveillance-yyyy.csv” (where “yyyy” is the year of monitoring), is described in Table 5. The majority of equids’ infections were detected in Veneto and Lombardia, where horse breeding is more frequent in the territory. A substantial decrease in West Nile cases in horses is registered in Veneto after 2019, perhaps highlighting the effect of a wide campaign of prevention carried out by the region to raise awareness among horses’ owners. The highest number of horses’ infections in Lombardia is instead recorded in 2019 and 2020.

**Table 5:** Structure of the file “wn-ita-equids-surveillance-yyyy.csv” within the folder “equids-surveillance”.

Variable	Description	Format
url_bulletins	Link to the bulletin in PDF format	String
data	Week reference date	yyyy-mm-dd
code_region	Region 2-digit code	Numeric
name_region	Region name	String
code_province	Province 3-digit code	Numeric
name_province	Province name	String
abbreviation_province	Province 2-letter code	String
lat	Latitude of the province	Numeric
long	Longitude of the province	Numeric
new_cases	Total amount of new cases (“total_cases” current date - “total_cases” previous date)	Numeric
total_cases	Total number of cases	Numeric
new_deaths	Total amount of new deaths (“total_deaths” current date - “total_deaths” previous date)	Numeric
total_deaths	Total number of deaths	Numeric
num_equids_outbreak	Total equids present in the outbreak	Numeric

### Birds surveillance data

This folder contains weekly cases of WNV infections in birds at the province level. For monitoring purposes, cases in birds are reported separately for target and wild species. The choice on the part of IZS-AM to monitor these two types of birds is as follows: wild species can act as reservoirs and amplifiers of viral infection, while target species are sedentary so they allow viral circulation to be determined in specific areas of regions. The folder includes two different .csv files, one for each type of bird: (i) the csv file “wn-ita-target-birds-surveillance-yyyy.csv” (where “yyyy” is the year of monitoring) has data about three targeted species, i.e., *Magpie* (*Pica pica*), *Gray crow* (*Corvus corone cornix*), and *Jay* (*Garrulus glandarius*); (ii) the csv file “wn-ita-wild-birds-surveillance-yyyy.csv” (where “yyyy” is the year of monitoring) reports WNV infections of the wild birds. Note that WNV has been found in up to 52 different wild bird species across the years, but this information was not reported for the 2021 monitoring. The structure of both csv files is reported in Table 6. Emilia-Romagna detected the largest number of birds’ infections in almost all years. This higher detection rate, if compared with other regions, can be due to a better application and adherence to the parameters of birds’ sampling in application to the regional Arbovirosis prevention law, and higher awareness of the regional governors.

**Table 6:** Structure of the files “wn-ita-target-birds-surveillance-yyyy.csv” and “wn-ita-wild-birds-surveillance-yyyy.csv” within the folder “birds-surveillance”.

Variable	Description	Format
url_bulletins	Link to the bulletin in PDF format	String
data	Week reference date	yyyy-mm-dd
code_region	Region 2-digit code	Numeric
name_region	Region name	String
code_province	Province 3-digit code	Numeric
name_province	Province name	String
abbreviation_province	Province 2-letter code	String
lat	Latitude of the province	Numeric
long	Longitude of the province	Numeric
species	Species name of reported cases	String
new_cases	Total amount of new cases (“total_cases” current date - “total_cases” previous date)	Numeric
total_cases	Total number of cases	Numeric

To facilitate access to the whole available data, we also created a unique dataset named “latest-wnv.csv” whose structure is reported in Table 7. The file has 12 columns and each row identifies the weekly cases of WVN infections by type of host at the province level. The idea was to provide researchers and practitioners with a compact version of the different surveillance data–excluding specific aspects, such as age and type of infection for human surveillance or species for bird surveillance–to allow for a quick comparison among cases by different type of host and to ease data visualization of such data to promptly identify the areas where the virus is more likely to spread in order to increase the effectiveness of prevention policies.

**Table 7:** Structure of the file “latest-wnv.csv”.

Variable	Description	Format
url_bulletins	Link to the bulletin in PDF format	String
data	Week reference date	yyyy-mm-dd
code_region	Region 2-digit code	Numeric
name_region	Region name	String
code_province	Province 3-digit code	Numeric
name_province	Province name	String
abbreviation_province	Province 2-letter code	String
lat	Latitude of the province	Numeric
long	Longitude of the province	Numeric
new_cases	Total amount of new cases (“total_cases” current date - “total_cases” previous date)	Numeric
total_cases	Total number of cases	Numeric
host	Type of infected host	String

## Technical Validation

Due to delays in reporting and possible measurement errors, computation of WNV weekly cases as first differences of cumulative cases may yield negative values. These negative values are clearly artificial and should not be present in the data. Sadly enough, this is not only an issue of WNV surveillance data, but a common drawback of (almost) all cumulative counts of epidemiological indicators^30^. Indeed, as it will be also discussed later, cumulative counts are subject to a monotonicity constraint and, subsequently, new counts (a.k.a. innovations) must be nonnegative. Therefore, all negative values of weekly cases (“new_cases”) have been set as “Not Available” (“NA”) whenever the current week’s value of cumulative cases (“total_cases”) was smaller than the value of “total_cases” in the previous week. We checked the occurrence of this issue and note that there are a total of 23 missing values in human cases of WNV at the province level, 9 missing values in human cases of WNV at the regional level, 3 missing values in equids cases of WNV, 5 missing values in mosquitoes cases of WNV and 15 missing values in birds cases of WNV. However, these are randomly distributed by monitoring week, type of infection (when applicable) and province, and represent less than 5% of the observations in all cases, therefore not inducing any substantial bias in the dataset. In addition, we checked and confirm that the remaining missing values in WNVDB are structural and due to the lack of information. Indeed, not all the information about the surveillance is available across years and for the different types of hosts. For example: the name of the province was not always indicated for neuroinvasive human infections, but reported as “Not indicated”, as well as age class; the total number of equids that were present in the outbreak (“num_equids_outbreak”) is missing for the monitoring week of September 26, 2018; the “code_region” is obviously missing for all cases imported from abroad, jointly with the geo-location, the birds species is missing throughout 2021, etc. In all these cases, while the single record may be less informative, it could still be used to aggregate data and may provide further insights into the understanding of WNV spread patterns. A possible solution to the missingness in the data could have been imputation using any valid statistical technique; however the imputation of epidemiological indicators is still an open research topic requiring further investigation^31,32^, and it is therefore out of the scope of this work.

Finally, to ensure that the digitalization process is robust and to check intra-record coherence and consistency, we select subsequent samples, each one composed of 100 observations from the outbreak records and manually verify that the information coincides with the related PDFs. In the case of a mismatch, data are corrected and the procedure is re-run until no mismatches are found between the digitalized files and the bulletins.

Eventually, to further prove the validity of WNVDB, we model the yearly WNV outbreaks in 2018 and 2022 for the two most affected Italian regions, i.e., Veneto and Emilia-Romagna using the Richards’ curve^33^. This curve, also known as Generalized Logistic Function, is very flexible and allows to characterize the main features of an epidemic outbreak (e.g., peak time, carrying capacity, etc) and to provide short-term forecasts of WNV evolution. This proposal already proved successful in modelling other epidemic outbreaks, such as the one of SARS, Dengue, Zika, Monkeypox and Ebola^34–39^, for which similar surveillance plans and data collection processes were implemented.

### Richards’ growth generalized linear model

Cumulative incidence of severe epidemic outbreaks typically exhibits an S-shaped trend: the onset of the outbreak anticipates an exponential growth phase whose acceleration usually softens after the implementation of one or more prevention policies (e.g., lockdown, vaccination campaign, etc.), eventually reaching an asymptote that can either be constant if the virus is eradicated or time-varying if the virus goes endemic. From another perspective, the first differences of such cumulative incidence indicators generally exhibit a bell-shaped behaviour (wave), still highlighting the different phases of the epidemic growth pattern. Borrowing from a well-known tool in the biological literature, we here use the Richards’ curve^33^ to model weekly cases of WNV at the regional level for the two most severe outbreaks of 2018 and 2022^24^. Nevertheless, we argue that this model specification can also be used to model smaller epidemic outbreaks in different regions. In particular, this curve is specified by 5 parameters able to characterize most of the features occurring in epidemiological data, such as the final epidemic outbreak size, the infection growth rate, the peak position (i.e., when the curve growth speed slows down) and the slopes of the ascending and descending phase of the outbreak^40^.

For each year and for a given spatial aggregation level, let us denote the weekly time-series of cumulative West Nile cases by ${\left\{{y}_{t}^{c}\right\}}_{t=1}^{T}$. We model the expected value of the cumulative counts independently for each year and geographical unit by assuming that its expected value follows the *extended* Richards’ curve^33,41^, which can be defined as:

1 $${\mathbb{E}}[{Y}_{t}^{c}]={g}^{-1}(t;{\boldsymbol{\gamma }})={\lambda }_{{\boldsymbol{\gamma }}}(t)=b\cdot t+\frac{r}{{(1+{e}^{h(p-t)})}^{s}},$$

where γ^*T*^ = [*b*, *r*, *h*, *p*, *s*] is the 5 parameters vector including: *b* ∈ R^+^ and *b* · *t* represents a linear trend on the baseline, *r* > 0 the distance between the upper and the lower asymptote, *h* the *hill* (growth rate), *p* ∈ (0, *T*) the inflation point, and *s* ∈ R the asymmetry parameter. Note that each cumulative count can be defined as:

$${Y}_{t}^{c}=\mathop{\sum }\limits_{\tau =0}^{t}{Y}_{\tau }\quad \Rightarrow \quad {Y}_{t}={Y}_{t}^{c}-{Y}_{t-1}^{c},\quad \quad t=1,\ldots ,T$$

where *Y*_0_ = 0 without loss of generality. In other words, no matter the time scale, cumulative counts at time *t* are obtained by summing all the new counts from the beginning of the monitoring period up to *t*. Looking at this relationship from the opposite side, new counts at each time *t* are the result of the difference between cumulative counts at time *t* and *t* − 1. Exploiting the linearity property of the expected value, from (1) we can easily derive the expected value of new counts at each time point as:

2 $${\mathbb{E}}[{Y}_{t}]={\mathbb{E}}[{Y}_{t}^{c}]-{\mathbb{E}}[{Y}_{t-1}^{c}]={\lambda }_{{\boldsymbol{\gamma }}}(t)-{\lambda }_{{\boldsymbol{\gamma }}}(t-1)={\widetilde{\lambda }}_{{\boldsymbol{\gamma }}}(t)=b+r\cdot [{(1+{e}^{h(p-t)})}^{-s}-{(1+{e}^{h(p-t+1)})}^{-s}].$$

From (2) is even more clear that the parameter *b* can be interpreted as the constant endemic rate. We do so to allow for the direct modelling of new counts instead of cumulative ones. It is indeed not trivial to specify a suitable probability distribution that ensures the natural constraint they must respect, i.e., *Y*_*t*_ ≥ *Y*_*τ*_, ∀*τ* ≤ t. On the other hand, by assuming stochastic independence, conditionally on *λ*_*γ*_(*t*), between the new counts at the time *t* and all the previous observed values, i.e., ${Y}_{t}\left|{\boldsymbol{\gamma }}\perp {Y}_{\tau }^{c},\forall \tau  < t\right.$, we can easily express the likelihood of any suitable probability distribution for count data, such as the Poisson or the Negative Binomial. Embedding Richards’ specification in the expression of the expected value of the observed counts defines an extended GLM framework that is flexible enough to model several growth curves, such as one of WNV cases. Parameters are estimated by numerical maximization of the log-likelihood as analytical solutions are not available in closed form. Further insights on the model implementation and estimation are provided in the seminal paper^42^, as well as additional details on how to compute the forecast and related uncertainty.

**Fig. 3:**
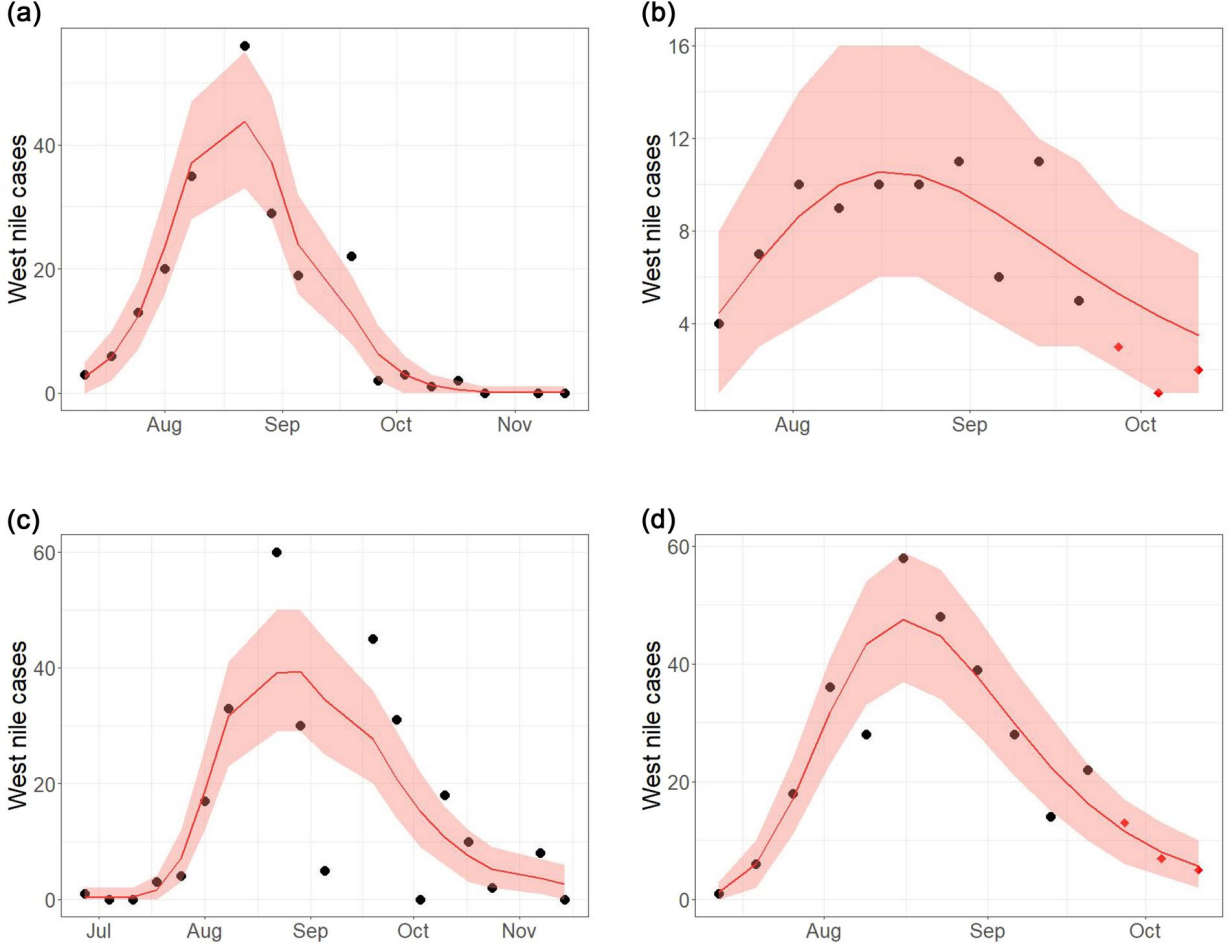
Observed data (black dots) and estimated Richards’s curves (solid red line) with 95% confidence intervals (red shaded area) of West Nile outbreaks for (**a**) Emilia-Romagna in 2018, (**b**) Emilia-Romagna in 2022, (**c**) Veneto in 2018 and (**d**) Veneto in 2022. Red dots represent the pointwise forecasts for the outbreaks in 2022.

Richards’ parameter estimation is summarized in Table 8. Looking at the results of the model for the 2018 outbreak, it is possible to highlight a satisfactory goodness-of-fit for both regions, with *R*^2^≈0.91 and *R*^2^≈0.61 for Emilia-Romagna and Veneto, respectively (see left panels of Fig. 3). As clear from Figure 3c,the management and collection of the data in Veneto is very heterogeneous, and this would affect the uncertainty surrounding our estimates. Data issues are well-known and widely discussed in the literature^30^. However, the Richards’ curve is able to capture the average outbreak behaviour. In detail, the estimated final epidemic size of the outbreaks in 2018 is equal to 0.4417 (CI_0.95_ = [0.4396, 0.444] and 0.9104 (CI_0.95_ = [0.9103, 0.9105] infections each 1000 residents in Emilia-Romagna and Veneto, respectively, with Veneto experiencing a more at-risk situation. This is not the only difference between the two regions, as the spread of the epidemic follows a smoother behaviour in Veneto than in Emilia-Romagna (see Figure 3a), with the parameter governing the infection growth rate being $\widehat{h}=0.8082$ (CI_0.95_ = [0.8081, 0.8083] and $\widehat{h}=0.4084$ (CI_0.95_ = [0.4084, 0.4085]. At the same time, however, the decreasing phases is also smoother and slower in Veneto than in Emilia-Romagna, leading to a higher overall outbreak size. The idea that Veneto is more affected than all other Italian regions is confirmed by the analysis of the 2022 outbreak. On the other hand, the estimated final epidemic size of the more recent outbreaks in 2022 is equal to 0.30 (CI_0.95_ = [0.019, 0.87] and 0.5842 (CI_0.95_ = [0.5842, 0.5842] infections each 1000 residents in Emilia-Romagna and Veneto, respectively. The goodness-of-fit is still acceptable and consistent over outbreaks with *R*^2^≈0.56 and *R*^2^≈0.84 for Emilia-Romagna and Veneto. As clearly shown in Figure 3b, the size of the outbreak in Emilia-Romagna is much smaller and small changes in the number of cases could lead to a more uncertain estimate of the overall outbreak size. Given the different sizes, the characteristics of the region-specific outbreaks are rather similar to those from 2018. To remark that the Richards’ curve can be used not only to describe the outbreak but also to obtain short-term forecasts, we estimate the outbreak behaviour leaving out the last three observations and then check if they are included in our forecast intervals. For both regions, we are able to forecast short-term events with a reasonably small uncertainty. This result can be used for future outbreaks, to monitor the evolution of the outbreak and to timely plan interventions, if required. Note that the same approach is valid for the spread of the WNV among animals too, as a further justification of the usefulness of our proposal.

**Table 8:** Point estimates (95% confidence interval) of the Richards’ parameters.

Region	Year	*r*	*h*	*p*	*s*	*b*
**Emilia-Romagna**	2018	0.2517 (0.2505, 0.253)	0.8082 (0.8081, 0.8083)	5.4322 (5.4318, 5.4326)	0.0553 (0.055, 0.0556)	0.190 (0.1891, 0.191)
**Veneto**	2018	0.0636 (0.0636, 0.0636)	0.4084 (0.4084, 0.4085)	−16.4633 (−16.9927, −15.934)	10 (9.7844, 10.2156)	0.8468 (0.8467, 0.8469)
**Emilia-Romagna**	2022	0.04 (0.005, 0.38)	0.2537 (0.2531, 0.2543)	−20.29 (−28.69, −11.90)	6.37 (4.25, 8.49)	0.26 (0.014, 0.59)
**Veneto**	2022	0.165 (0.165, 0.165)	0.3909 (0.3909, 0.3909)	−3.37 (−3.38, −3.36)	3.486 (3.484, 3.489)	0.4192 (0.4192, 0.4192)

## Usage Notes

WNVDB is designed to promote rapid and objective epidemiological reading of available data. This allows, for instance, monitoring the epidemiological trends of the West Nile outbreaks in Italian regions and provinces with informative graphical outputs and deriving insights with predictive modelling. Recall that this data descriptor was peer-reviewed in 2023 based on the data available on Zenodo platform at the time, i.e., with data up to November 2022^29^. However, to facilitate other research works and ease the utilization of WNVDB, we have deposited metadata and R codes in a GitHub repository (https://github.com/fbranda/west-nile), which is released under a Creative Commons Attribution 4.0 International (CC BY 4.0) license, allowing users to share, copy and redistribute the material in any medium or format and to adapt, remix, transform and build upon it for any purposes. At the same link, we are hosting the dynamic version of WNVDB that will be continuously updated (and eventually adapted) as soon as new bulletins are published. The same technical procedures for data extraction and validation discussed in this paper will be applied to any new event added to the dataset. Usually, every year, the monitoring period of West Nile cases ranges from the end of May to November. Within the GitHub interface, users have the capability to mark potential inaccuracies in the dataset. This action will initiate an error correction procedure that primarily involves subjecting the reported record to an additional validation cycle to verify and replace any erroneous fields, if necessary. Through the WNVDB GitHub repository, our primary aim is to foster collaboration and engagement among specialists in the field. By providing a centralized platform for sharing datasets, analyses, and findings related to WNV, we hope to catalyze advancements in understanding the virus’s epidemiology, transmission dynamics, and potential interventions. This repository not only promotes transparency and reproducibility in research but also serves as a valuable resource for researchers, public health professionals, and policymakers striving to combat the virus’s impact. Finally, in collaboration with the Agency for Digital Italy (AgID), we also hosted the dataset into their official website (https://tinyurl.com/agid-west-nile). For interested readers, we mention that WNVDB data at the province level can be easily joined with environmental factors obtained from the World Weather Online dataset (https://www.worldweatheronline.com/). This demonstrates the interoperability of our dataset with other official data sources and shows how to assess possible relationships between the spread dynamics and the environmental conditions. In particular, the literature suggests that the mean maximum temperature, the mean total precipitation and the mean wind speed affect WNV transmission in different contexts^43–48^. Our goal is to reach as many people as possible, from the individual citizen to the professional, to evolve the current Italian open data portal into a system that provides tools and services developed and shared with the community to extend its potential.

## Data Availability

Refer to the README file accessible at the GitHub repository (https://github.com/fbranda/west-nile) for further instructions on how to use the dataset, import it either in R or Python, and carry out some exploratory analysis. The same link also hosts the dynamic version of WNVDB and all source codes to reproduce the results reported in this Data Descriptor.
